# Treatment Protocols for Eating Disorders: Clinicians’ Attitudes, Concerns, Adherence and Difficulties Delivering Evidence-Based Psychological Interventions

**DOI:** 10.1007/s11920-016-0679-0

**Published:** 2016-02-18

**Authors:** Glenn Waller

**Affiliations:** Clinical Psychology Unit, Department of Psychology, University of Sheffield, Western Bank, Sheffield, S10 1NT UK

**Keywords:** Eating disorders, Evidence-based practice, Protocols, Manuals, Adherence therapist drift

## Abstract

There are several protocols in existence that guide clinicians in the implementation of effective, evidence-based psychological interventions for eating disorders. These have been made accessible in the form of treatment manuals. However, relatively few clinicians use those protocols, preferring to offer more eclectic or integrative approaches. Following a summary of the research that shows that these evidence-based approaches can be used successfully in routine clinical settings, this review considers why there is such poor uptake of these therapies in such settings. This review focuses on the role of service culture and on clinicians’ own attitudes, beliefs and emotions. Possible means of enhancing uptake are considered, but these cannot be considered to be ideal solutions at present.

## Introduction

Protocols, usually codified in the form of treatment manuals, are key methods for the dissemination and reliable implementation of evidence-based psychological treatments. It is important to recognise a protocol for what it is intended to be—a broad set of methods, designed to be applicable to the patient in a way that is reflective of their individual case, but guided by a set of principles. The clinical skill and artistry in the delivery of evidence-based therapy lie in how the clinician implements the protocol for the individual patient (1). However, despite this clear recognition that protocols and manuals need to be used flexibly, they are perceived negatively by many clinicians (2), who regard them as constraining their practice and artistry by limiting the individualisation of formulation and intervention approaches (1). Therapists routinely deviate from those evidence-based approaches—a phenomenon termed ‘therapist drift’ (3, 4).

Many clinicians argue that *evidence-based practice* is a more useful approach than the use of empirically supported interventions. Evidence-based practice combines those empirically-supported approaches (usually based on the use of a protocol/manual) with clinicians’ own judgement and patients’ values. However, there are two issues to consider here. First, the evidence to date does not really support the superiority of this wider ‘evidence-based practice’ approach. In most cases, clinician judgement results in poorer outcomes than protocol-based approaches (5, 6), and there is little evidence that the individualised formulations that clinicians develop are clinically reliable or useful to the patient (7). Second, in eating disorders in particular, patient values are often in direct conflict with the necessary elements of evidence-based treatments—for example, the anorexia nervosa sufferer who wants to achieve recovery (e.g. regaining their health, completing their education, being able to have children, being accepted by those around them) whilst remaining at a weight that makes this impossible, or the bulimia nervosa sufferer who wants treatment that alleviates their bulimic symptoms whilst losing weight at the same time. Therefore, one needs to be wary of the assumption that evidence-based practice (with its tripartite nature) will be superior to the single element of a protocolised evidence-based treatment.

Clinicians working with eating disorders are potentially fortunate, in that there exists a range of evidence-based protocols that we can use to guide our clinical practice (8–14, 15•, 16, 17). Whilst none of these achieves 100 % success (see below), they have demonstrable levels of benefit and can be implemented in routine clinical practice. This review will briefly consider the evidence that we can treat the eating disorders in research and routine clinical settings, using protocolised approaches. It will then explore whether we use those approaches in routine practice and the reasons that we do or do not do so. Finally, ways in which clinicians might be encouraged to use evidence-based protocols and methods and whether such encouragement is likely to be effective will be considered.

### The Evidence Base for Psychological Treatments for Eating Disorders

Once a protocol has been developed and piloted, evidence-based treatment requires two major forms of study before it can be considered to be viable. First, there need to be well-controlled trials of the protocol in research settings (‘efficacy studies’). Then, the treatment needs to be tested in routine clinical settings (‘effectiveness studies’). Both have been carried out in the field of treating eating disorders.

### Efficacy Studies

There is clear evidence from large research trials that some therapies are efficacious in working with eating disorders. That evidence had been well summarised up until a few years ago, in the form of a number of reviews with very consistent conclusions (18–24). A reasonable summary of those reviews might have led to the following summary, a decade ago:

- A number of therapies had been identified that reduced the severity and presence of bulimia nervosa and binge-eating disorder, including cognitive-behavioural therapies (CBT), interpersonal psychotherapy (IPT), and dialectical behaviour therapy (DBT). Of those therapies, CBT had the strongest evidence of the greatest and most rapid effects.
- Those therapies were usually more effective if delivered in an individual, face-to-face setting.
- Medications had a limited role in the treatment of eating disorders, with some evidence for symptom alleviation in bulimia nervosa and binge-eating disorder, but no support for treating anorexia nervosa and no clear evidence for medication/psychotherapy combinations being superior. The studies were often hard to rely on as indicators of remission or recovery, as they had very short follow-ups.
- Younger adolescents with less long-lasting cases benefitted more from family-based therapies (FBT).
- Treatments for adults with anorexia nervosa had weak evidence, with weak outcomes (even when they include treatments delivered in more intensive settings), and little difference between therapies.
- There was virtually no evidence base relating to the treatment of atypical eating disorders (other than binge eating disorder), despite this category representing the largest single category of eating disorders (19).

Since those reviews were published, a number of important treatment trials have been published in the field. The key changes in our evidence base as a result can be summarised as:

- CBT has been shown to be effective in the treatment of atypical cases of eating disorder where the patient is not underweight (25), though there is no evidence that there has been any improvement in outcomes for bulimia nervosa or binge eating disorder with developments in CBT.
- Outcomes for patients with anorexia nervosa are somewhat improved, particularly for CBT (26••, 27), though not in modified versions of CBT where weight gain is de-emphasised (28). CBT is now demonstrably more effective than other therapies for this group, at least by the end of therapy (29, 30). Its effects remain weak compared to the outcomes for patients who are not underweight, with recovery in only approximately 30 % of anorexia nervosa patients entering CBT, but this is better than the outcomes demonstrated by other therapies (15•, 30, 31).

Other findings in recent years (32) simply reinforce the conclusions from the earlier literature, as outlined above. It is particularly noteworthy that there has been little improvement in the outcomes of pharmacotherapy in that time (33). In particular, it cannot be assumed that combining medication with psychotherapies is a useful strategy, as the combination does not reliably add to the benefits of the psychotherapies in isolation (34).

Finally, it is worth noting that the change in diagnostic criteria with DSM-5 does not appear to have altered outcome rates appreciably. However, it does mean that there is some doubt about the comparability of studies.

### Effectiveness

Obviously, it is important to consider whether these evidence-based treatments can be delivered outside of highly resourced research settings. A common belief amongst clinicians is that the results from such research trials cannot be replicated in routine clinical settings, due to the patients in research settings being more carefully selected (e.g. to exclude comorbidity), an unachievable level of supervision and training for therapists, and greater resources being spent on the treatment than in routine practice. However, this is an empirical question—can the results from clinical research (efficacy) be replicated in everyday practice (effectiveness)?

Fortunately, the past decade has seen the publication of several effectiveness studies that give a clear answer to this question, using large routine clinical populations of patients with different diagnoses (35–40), and the answer is clear. The clinical outcomes are very close to those found in research trials, though the attrition rate is somewhat higher. Unfortunately, the literature is almost entirely based on CBT. Therefore, there is a need for more effectiveness studies to determine whether other evidence-based therapies maintain that evidence in everyday practice. However, given the lack of support for common clinician beliefs (e.g. ‘The research just does not apply here’) relating to the most strongly evidenced psychological therapy, one might argue that clinicians who want to discount other therapies should be required to make the case that the evidence base cannot be applied in their place of work.

### How Widely Used Are the Evidence-Based Interventions?

Despite the evidence outlined above, the simple answer to this question is: ‘Not very widely’. The number of eating-disorder specialist clinicians who report adhering to evidence-based protocols and manuals is between 6 and 35 % (41, 42). Far more clinicians report that they use (un-tested or un-supported) mixtures of some techniques that are derived from empirically supported treatments and some techniques that are not supported even at that level (41, 43).

Even when clinicians say that they are using an evidence-based therapy, that claim should be treated with caution. For example, many clinicians who state that they are delivering CBT or FBT report that they omit many of the key techniques that make up those treatments (44, 45, 46•, 47). This omission is reflected in the accounts of eating-disordered patients reporting on the treatment that they have received (48•, 49), as their accounts suggest widely divergent patterns of techniques delivered under the title of CBT.

A further complication is that the same label is used for therapies with different content. CBT is a classic example of this issue, with the content differing in important ways. For example, one might not be surprised that a form of CBT for anorexia nervosa that reduced or removed the emphasis on weight gain (the key outcome variable [21]) has relatively low rates of weight gain (28). Similarly, in recent years, there has been widespread dissemination of an enhanced form of CBT (CBT-E [10]), though it has never been compared directly with the previous form of CBT to determine whether it is more effective with bulimia nervosa or binge eating disorder. Indeed, one has to be careful to understand which version of CBT-E is being used when considering outcomes, as the original two versions (broad and focused) have converged into a single version that combines the focused form with the affect regulation module of the broad form (50). Finally, studies vary in how they treat outcomes, with some studies of anorexia nervosa treating hospitalisation as a routine event and others as a reason for defining therapy as having failed (26••, 29). Whilst developments and differences in delivery are clinically understandable, the key differences need to be highlighted, to ensure that differences in outcomes can be understood.

To summarise, the number of patients who receive evidence-based therapies is probably very low, outside of research settings. This pattern of therapist drift and the consequent evidence-practice gap are not confined to eating disorders (3, 4). However, as in other disorders, the gap is a concern because we have no idea whether it is justified in terms of patient benefits. Few clinicians document or report on the outcome of their eclectic or integrationist (or random) approaches to treatment. The use of individualised approaches to treatment of eating does not preclude measurement of effectiveness, but the lack of such measurement does not reassure others that this individual-centred approach is more effective than a protocol-based approach. Indeed, it is important to remember that an individual-centred approach based on clinician judgement has been shown to be substantially less effective than the use of a protocol-based approach (6). Therefore, once again, the obligation to demonstrate effectiveness could be argued to lie with the clinician who adopts the non-standard approach.

### Why Are Evidence-Based Therapies So Rarely Used for the Eating Disorders?

Difficulties with delivering evidence-based therapies can be identified at different levels. First, there are *service-centred reasons*. Eating disorder services vary in their culture, and consequently, their willingness to change towards evidence-based methods. Possibly the best example of service-level reluctance is that provided by Lowe and colleagues (51), who attempted to introduce a ‘normalisation of eating’ module into the care package delivered by an existing set of eating disorder services. They described extreme difficulties in introducing this element of treatment, facing resistance based on the staff teams’ and services’ philosophy and practice of care, even though the administrators of the clinical services had approved the proposed change.

Second, *patient-centred reasons* exist for non-use of evidence-based methods. Patients’ values can conflict with the delivery of evidence-based treatments. Many patients who have previously had therapy express a preference for an approach that is less challenging, omitting key elements such as weighing and record-keeping (52). Given the degree to which therapists omit some of the key elements of therapy (see above), it cannot be a surprise that many eating-disordered patients have previously had several unsuccessful therapies and have become acculturated to the idea that therapy is a relatively unchallenging experience. In short, inadequate therapy experiences have the potential to teach patients that treatment should not be challenging in any way, when it is clear that the evidence-based approaches are based on changing ingrained behavioural, social, cognitive and emotional patterns. Of course, this avoidance of challenging is not confined to the patient, as clinicians also seem to do the same thing in order to reduce their own anxiety (see below). This joint pattern is perhaps most clearly seen when the therapist and patient fall into the pattern of discussing causes of the eating disorder without engaging in change of the maintaining factors (e.g. not changing eating patterns).

Finally, there are *clinician-centred reasons* for non-delivery of evidence-based approaches. One key reason applies to psychological therapies in general—many clinicians (approximately one third) are untrained in the therapy that they are employed to deliver (53, 54). The second important reason is the fact that many clinicians’ actions typify the ‘affiliation hypothesis’—because one believes in a therapy’s effectiveness, it will be effective when one uses it. In the case of the eating disorders, this is a hypothesis that has been comprehensively disproved by the work of Poulsen and colleagues (55••), who have shown that therapists being affiliated to a psychodynamic approach to treating bulimia nervosa does not mean that their recovery rate comes even close to the outcome from evidence-based CBT, even when the psychodynamic therapy is several times longer.

Clinician beliefs and attitudes have a strong influence on clinical practice. Outside the field of eating disorders, it has been shown that clinicians’ ratings of their skills and clinical outcomes indicate that we believe we are far more effective that we actually are (56–58). There is no reason to assume that eating disorder clinicians differ from this general pattern of beliefs. Another clinician-based factor is our knowledge of and attitudes to protocols and manuals. These vary considerably from clinician to clinician (2, 59), within and outside the eating disorders. Some clinicians are unaware of the existence of manuals and protocols and others hold very negative attitudes towards them. It is also clear that we hold attitudes to some elements of therapy for eating disorders that are not in keeping with the evidence about them. For example, some elements of therapy for eating disorders appear to be overvalued, such as pre-therapy motivational work (60, 61) and the presumed universal importance of prioritising the development of a strong working alliance in order to facilitate change (62, 63). In contrast, clinicians undervalue some key elements of therapy for eating disorders, such as the need to weigh patients regardless of the specific evidence-based model (64). Finally, clinicians often hold beliefs about what Meehl (5) terms ‘broken leg exceptions’—leading us to exclude patients from evidence-based treatments on the grounds of apparently spurious ‘justifications’. Those ‘justifications’ can include patient chronicity, comorbidity and complexity, even though the evidence would not support these as reasons for such an exclusion or change in practice (65–67).

Clinicians’ emotions are also important in understanding the delivery of protocols and evidence-based treatment. As with other disorders (68•), clinicians who are more anxious are less likely to deliver some of the key elements of treatment for eating disorders (44, 47). This avoidance of more ‘demanding’ elements of therapy has been suggested to be an example of clinicians engaging in their own safety behaviours—avoidance of tasks (e.g. weighing patients; changing their food intake). Such avoidance means that the clinician does not feel like a ‘bad therapist’ for distressing the patient, even though the longer-term result is that the patient does not improve and the clinician is less effective (4). Meehl (69) has referred to this ‘protection’ of patients from the demands of change as reflecting clinicians having a ‘spun glass theory of the mind’ of their patients—the belief that our making demands of our patients will somehow damage them (like a fragile spun-glass decoration), even though such protection means that patients cannot learn to change for the better.

### How Could Clinicians Be Encouraged to Use Evidence-Based Methods?

The mean length of time for research findings to enter into routine clinical practice is a (perhaps) startling 15–20 years (70). In some cases, this delay is a matter of ignorance, and in others, it is a product of resistance at a variety of levels. Whilst ignorance of the existence of methods to treat different disorders can be overcome relatively easily through didactic methods, that is not the same as teaching or persuading clinicians to use them and to do so appropriately.

Training clinicians is a challenging area (71). Commonly, a single teaching session of a few hours to two days is treated as ‘training’, even though there is little evidence that this works. For example, whilst it is known that such sessions can influence knowledge of and attitudes to exposure therapy, both for eating disorders and anxiety disorders (72, 73), it is not yet known whether such changes translate into better use of the necessary skills in everyday practice. In short, does education translate into competence, and does competence translate into adherence to protocols and hence to more effective treatment? For example, many people have learned to drive safely whilst being closely monitored (competence), but after a short period of driving without that monitoring their driving deteriorates (non-adherence).

It is commonly assumed that clinical supervision is a means of ensuring that clinicians should adhere to best quality practice. However, there are some worrying caveats to that assumption. First, does the supervisor have an adequate grasp of the necessary evidence and skills base to be able to teach the supervisee to be adherent? Given the time it takes for evidence to reach practice (70) and the number of clinicians who lack training in the therapy that they deliver (53, 54, 55••), adequate supervision is an optimistic assumption in many cases. Second, do supervisors appraise their supervisees’ skills accurately? Whilst this question has not been investigated in the field of eating disorders, it has been considered elsewhere, and the worrying conclusion is that supervisors substantially overrate their supervisees’ clinical skills (74), with the likely result that the therapist continues to drift off-protocol. Clearly, further research is needed to help us to understand whether or not we can trust in the value of supervision to ensure the delivery of protocolized, evidence-based therapies for eating disorders, but the evidence to date is not promising.

Of course, a strong means of persuading clinicians to change their practice should be to require them to attend to patient outcomes. The existing effectiveness studies (35–40) show that such outcomes are easily collected, and yet few services collect or respond to such information. In part, that poor response might be the product of a lack of research/audit skills or dedicated time, either of which could be rectified. However, evidence from outside the field of eating disorders (75) points to a more worrying fact—that clinicians vary substantially in their interest or willingness to attend to outcome data. If data are regarded as being irrelevant, inconvenient or challenging, then the danger is that clinicians will ignore rather than respond to them (76).

Where clinicians do attend to data, it is important that they should be encouraged to attend to the most clinically meaningful data. In the case of eating disorders, those key data appear to be those that demonstrate early change and symptom remission. There is clear evidence that behavioural and attitudinal change in the first few sessions of CBT is a critical indicator of subsequent improvement of eating disorder symptoms (77, 78, 79••, 80••). However, whilst it is necessary, early change is not sufficient in isolation. It is also important to target some features by the end of treatment (e.g. normalisation of body image, improvement in psychosocial function), to reduce the risk of relapse (81).

## Conclusion

There have been substantial advances in the treatment of eating disorders, based on the development and establishment of evidence-based approaches. Such approaches are codified in the form of manualised protocols (8–14, 15•, 16, 17), which can be used with high levels of success in routine clinical settings (35–40). Indeed, the rate of success with protocol-based approaches is likely to be higher than that with approaches that prioritise our clinical judgement (1, 6).

However, this review has identified a serious gap in our use of research to drive everyday practice when working with eating disorders. Clinicians use evidence-based approaches, manuals and protocols rarely (41–45, 46•, 47, 48•)—a failure that can be attributed to our lack of knowledge, negative attitudes and emotional characteristics (47, 82). At present, a reliance on training and supervision to ensure competence and adherence is probably best described as an act of faith, requiring more clarity about what the training and supervision should contain. Our patients would benefit if clinicians and supervisors were to focus on clinical outcomes and the better implementation of protocols, to improve the level of patient improvement and recovery.
